# Exploring the dynamics and interplay of human papillomavirus and cervical tumorigenesis by integrating biological data into a mathematical model

**DOI:** 10.1186/s12859-020-3454-5

**Published:** 2020-05-05

**Authors:** Wenting Wu, Lei Song, Yongtao Yang, Jianxin Wang, Hongtu Liu, Le Zhang

**Affiliations:** 1 College of Computer Science, Sichuan University, Chengdu, 610065 China; 2 Department of Obstetrics and Gynaecology PLA General Hospital, Beijing, 100853 China; 3 BGI-Shenzhen, Shenzhen, 518083 China; 4 School of Information Science and Engineering, Central South University, Changsha, 410083 China; 5 National Institute for Viral Disease Control and Prevention, Chinese Center for Disease Control and Prevention, Beijing, 102206 China; 6 Medical Big Data Center of Sichuan University, Chengdu, 610065 China

**Keywords:** Cervical tumorigenesis, Human papillomavirus, Cluster analysis, Poisson regression

## Abstract

**Background:**

Cervical cancer is the fourth most common tumor in women worldwide, mostly resulting from high-risk human papillomavirus (HR-HPV) with persistent infection.

**Results:**

The present discoveries are comprised of the following: (i) A total of 16.64% of the individuals were positive for HR-HPV infection, with 13.04% having a single HR-HPV type and 3.60% having multiple HR-HPV types. (ii) Cluster analysis showed that the infection rate trends of HPV31 and HPV33 in all infections as well as HPV33 and HPV35 in single infections in precancerous stages were very similar. (iii) The single/multiple infection proportions of HR-HPV demonstrated a trend that the multiple infections rates of HR-HPV increased as the disease developed.

**Conclusions:**

The HR-HPV prevalence in outpatients was 16.64%, and the predominant HR-HPV types in the study were HPV52, HPV58 and HPV16. HR-HPV subtypes with common biological properties had similar infection rate trends in precancerous stages. Especially, as the disease development of precancer evolved, defense against HPV infection broke, meanwhile, the potential of more HPV infection increased, which resulted in increase of multiple infections of HPV.

## Background

Cervical cancer is the fourth most common tumor in women worldwide [1], mostly resulting from human papillomavirus (HPV). The current report [2] estimates that cancer cases related to HPV infection account for 4.5% of the total number of new cancers worldwide, of which cervical cancer accounts for 83% of these HPV infection-related cancers, posing a great threat to women’s health, especially in developing countries. Thirteen HPV genotypes denoted as high-risk HPV (HR-HPV) are essential factors for cervical tumorigenesis [2], so the dynamics of the HPV genotypes described here reflect the relationship between an individual HPV genotype and the development of cervical cancer, i.e., precancerous stages. Since it takes approximately 20 years for the carcinogenesis of HR-HPV with persistent infection [3], cervical cancer is the only malignant tumor that can be prevented and treated early through HPV-type screening, which plays a significant role in improving the prognosis of patients [3–5]. As China has become one of the countries with a high incidence of cervical cancer and HPV infection is widespread in females [6], it is very important to investigate HPV-type infections for Chinese population.

Generally, both the liquid-based cytology test (LCT) and the ThinPrep cytology test (TCT) are used to screen cervical cancer, but they do not effectively detect specific HR-HPV genotypes in infections. It is noted that we usually use the commercial names of LCT and TCT to represent the cytology tests, since they are from different manufacturers. However, HPV genotyping can easily detect the genotypes better than the LCT and TCT methods, and distinguish the difference between single infection (Denoted by Table 1) and multiple infections (Denoted by Table 1), once HPV infection occurs. Since the handicap of using cytology tests or HPV genotyping alone, we always employ the combination of cytology tests and HPV genotyping based on the significantly high sensitivity and lower false-negative rates achieved [7–11]. For example, Catteau et al. [12] calculated the prevalence rates of 13 HR-HPV-type infections in different precancerous stages among Belgian women, and Ying et al. [13] mainly employed the prevalence rates of different HR-HPV types in all precancerous stages to describe the distribution of the major infectious types in Beijing China after collecting the related data by both LCT/TCT and HR-HPV genotyping methods. Nevertheless, reported by the previous studies [12, 13], the prevalence of each HR-HPV type are different in the same precancerous stage. And for the same kind of HR-HPV type, the prevalence is not consistent in different precancerous stages [13]. Therefore, it is inaccurate to employ the total precancerous stage data to describe the relationship between HR-HPV types and precancerous stages.

**Table 1:** Nomenclature

Terms or equations	Description
All infections	It includes all subjects with HPV infections containing a specified HR-HPV genotype
Single infection	It refers to such infection with one HR-HPV genotype alone
Multiple infections	It refers to the difference between all infections and single infection for a HR-HPV type, i.e., HPV infected with two or more HR-HPV genotypes
All_Infection_Set	The dataset for all infections numbers of the 13 HR-HPV types in the four precancerous stages (see Additional file 1)
Single_Infection_Set	The dataset for single infection numbers of the 13 HR-HPV types in the four precancerous stages (see Additional file 1)
Multiple_Infection_Set	The dataset for multiple infections numbers of the 13 HR-HPV types in the four precancerous stages (see Additional file 1)

Furthermore, it is still unclear whether multiple infections are more risky than single infection of HR-HPV [13–15]. For example, Chaturvedi et al. [14] investigated the coinfection patterns of 25 HPV genotypes and computed the odds ratios for each genotype with 24 other genotypes. The results showed that the disease risk of multiple infections is close to the total estimated risk of individual infections. However, both Ying et al. [13] and Dickson et al. [15] indicated that women with multiple infections have a significantly higher risk of cervical disease than women with single infections. Since previous research collect data with different HPV types, patient ages and other related factors [13–15], they result in inconsistent conclusions for the risk of cervical lesions caused by multiple infections [16, 17]. Moreover, most previous studies [13–15, 18] employed cohort analysis for cervical cancer without considering the proportion of single and multiple infections in different precancerous stages for different HPV types as well as the impact of HR-HPV genotypes and precancerous stages on the infections.

Regarding to the previous shortcomings, we develop three innovations to overcome them: (1) we collected the clinical data for 13 HR-HPV types in 4 precancerous stages by integrating TCT into HR-HPV genotype detection; (2) we performed cluster analysis for 13 genotypes in 4 precancerous stages; (3) we investigated the proportion of single/multiple infections at 4 precancerous stages for each HR-HPV genotype, and explore the impact of HR-HPV genotypes and precancerous stages on the infections by Poisson regression [19].

A total of 16,693 patients were studied from July 2016 to July 2017 in the outpatient department of the General Hospital of the People’s Liberation Army. We first statistically analyzed infection data for 13 HR-HPV types in 4 precancerous stages. The results showed that the overall prevalence rate of the 13 HR-HPV types (16.64%) is less than the previous, but HPV52, HPV58 and HPV16 still have the greatest impact on the health of women in China. Next, we found that biological homology results in similar infection rate trends in precancerous stages by our k-means [20] cluster analysis. Finally, we not only found that the multiple infection proportion of HR-HPV increased as the disease developed, but also demonstrated that only the precancerous stages were statistically significant [19] by considering the impact of both HR-HPV genotypes and precancerous stages on infection results. Finally, we discuss the limitations and future study.

## Methods

### Pathological examination

Cervical cells were detected by TCT and the results of cytological pathology were diagnosed by senior physicians according to the Bethesda System of cervical cytology [21]. The precancerous stages are classified as follows [21]: (1) Normal; (2) Atypical squamous cells of undetermined significance (ASC-US); (3) Low-grade squamous intraepithelial lesions (LSIL); and (4) High-grade squamous intraepithelial lesions (HSIL).

### Detection of HPV genotypes

Thirteen HR-HPV genotypes (HPV16, HPV18, HPV31, HPV33, HPV35, HPV39, HPV45, HPV51, HPV52, HPV56, HPV58, HPV59 and HPV68) were detected with the real-time polymerase chain reaction kit for high-risk HPV genotypes from Shanghai ZJ biotechnology Company (http://www.liferiver.com.cn/productinfor/p15_62.html). The specific steps are strictly in accordance with the instructions of the kit. If the viral load of HPV-DNA was greater than or equal to 10^4^ copies/ml, it was positive, otherwise it was negative.

### Research subjects

The data in this study are from 16,693 patients who all underwent biopsies in the outpatient department of the General Hospital of the People’s Liberation Army from July 2016 to July 2017. The cervical samples were collected and detected with Riverlife Bio kits (http://www.liferiver.com.cn/productinfor/p15_62.html). Here, we have 15,706 Normal, 785 ASC-US, 69 LSIL and 133 HSIL cases. Also, we conducted quantitative detection for 13 HR-HPV subtypes in 16,693 cases to diagnose specific infection of HR-HPV genotypes.

### Workflow of the study

**Fig. 1:**
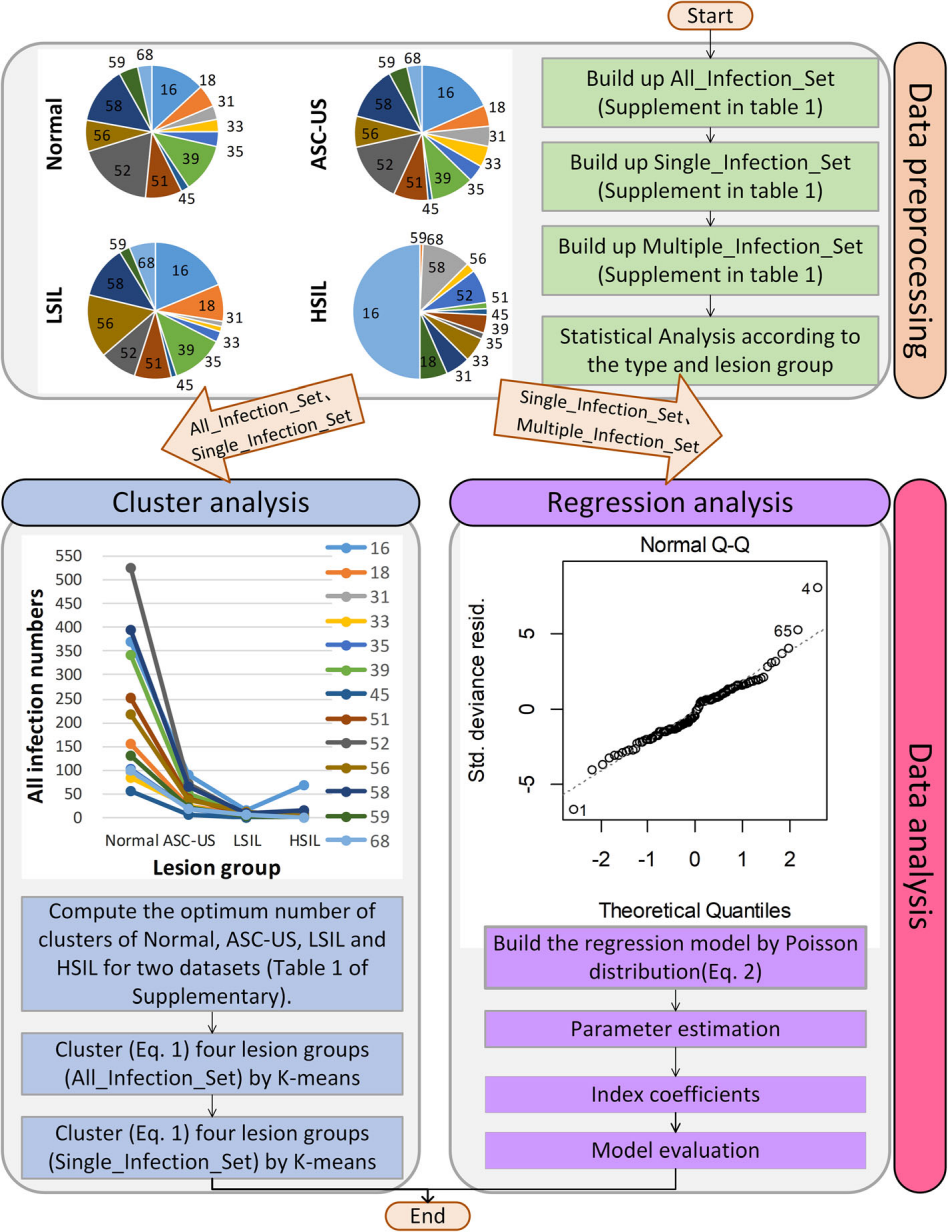
Workflow of the study

Figure 1 and Table 1 describe the workflow of the study and the nomenclature, respectively. The workflow consists of Data preprocessing and Data analysis steps. Data preprocessing step process the raw datasets for all infections (denoted by Table 1), single infection and multiple infections of the 13 HR-HPV types in the four precancerous stages by using a pie chart (left panel of data preprocessing component in Fig. 1) to describe the classical statistical analysis results.

Data analysis (Fig. 1) comprises cluster and regression analysis. Although the 13 HR-HPV genotypes are biologically independent, some of them may have common biological properties, resulting in similarity in the phenotypes (i.e., similarity in the number of infected people). Therefore, we use cluster analysis (left panel of data analysis in Fig. 1) to investigate the similarity of infection for 13 different HR-HPV types in precancerous stages. It is well known that the cluster analysis [22] consists of hierarchical clustering and nonhierarchical clustering. Since the aim of the study is to investigate which HR-HPV types have similar infections in the precancerous stages, we consider that the classical K-means [20] is suitable for this study. Here, K-means uses Euclidean distance (Eq. 1) [23] to measure the distance between two observed values:

1 $$ {d}_{ij}=\sqrt{{\left({x}_i-{x}_j\right)}^2} $$

*dij* represents the distance between observations of ith and jth HR-HPV genotypes. *xi* and *xj* represent the number of infected ith and jth HR-HPV genotypes, respectively.

Currently, Poisson regression is widely used for clinical data analysis [24]. For instance, Rochon et al. [25] used Poisson regression analysis to study the number of rejection reactions in patients after transplantation within a certain time, and Vonesh et al. [26] analyzed the potential risk factors related to the number of peritoneal bacterial infections. Here, we used Poisson regression (Eq. 2) to investigate the impact of HR-HPV genotypes and precancerous stages on infection [23].

2 $$ {\log}_e\left(\lambda \right)={\beta}_0+{\beta}_1{X}_1+{\beta}_2{X}_2 $$

Here, we set the infection number (*λ*) as the outcome variable and log_*e*_(*λ*) as the connection function in R software [27]. *X*_1_ and *X*_2_ respectively represent the HR-HPV genotypes and precancerous stages as the prediction variables. *β*_0_ is the intercept. *β*_1_ and *β*_2_ are coefficients for prediction variables.

## Results

### Comparison of the prevalence rates

**Fig. 2:**
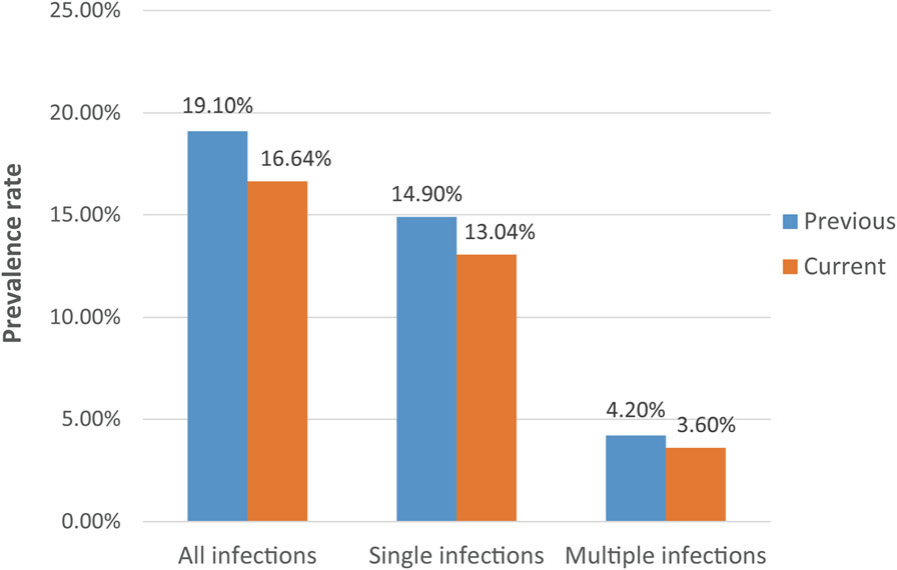
Current prevalence rate vs. previous prevalence rate

Figure 2 shows the comparison of the prevalence rates of the 13 HR-HPV types in all infections, single infection and multiple infections for the previous [13] and current study.

**Fig. 3:**
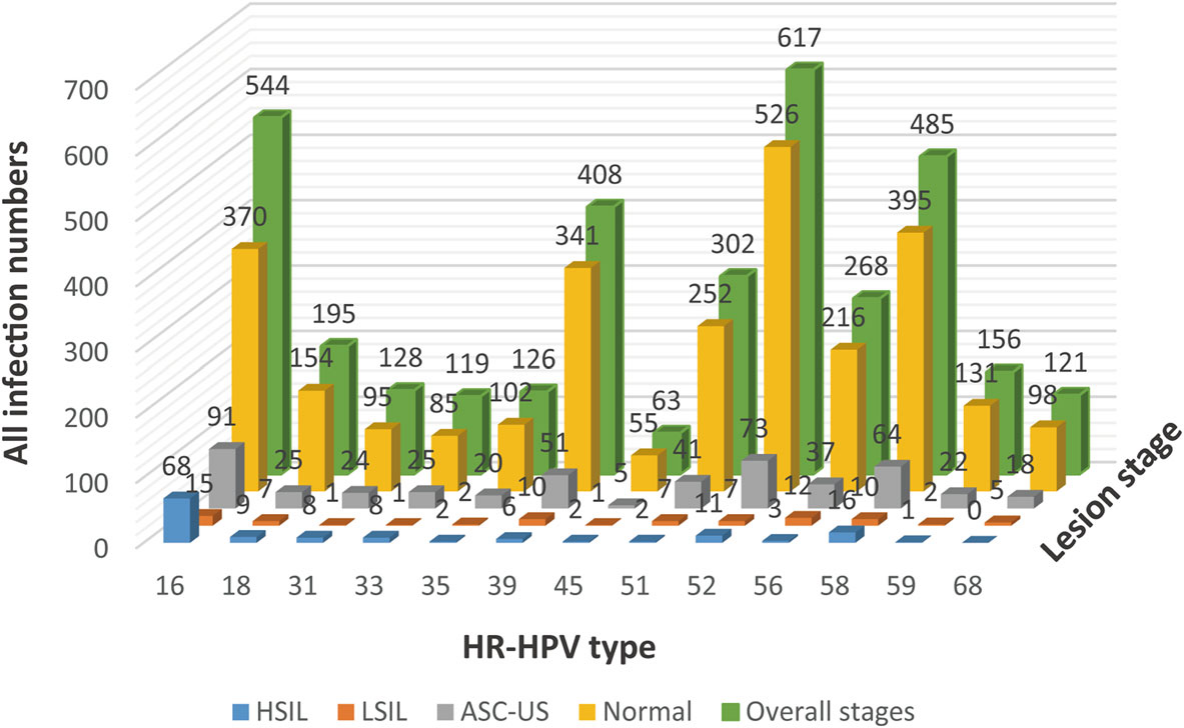
Infection numbers for different HR-HPVs and lesion groups

Figure 3 shows the dynamics of infections for each HR-HPV type at different precancerous stages.

### Cluster analysis of all infections and single infections

**Fig. 4:**
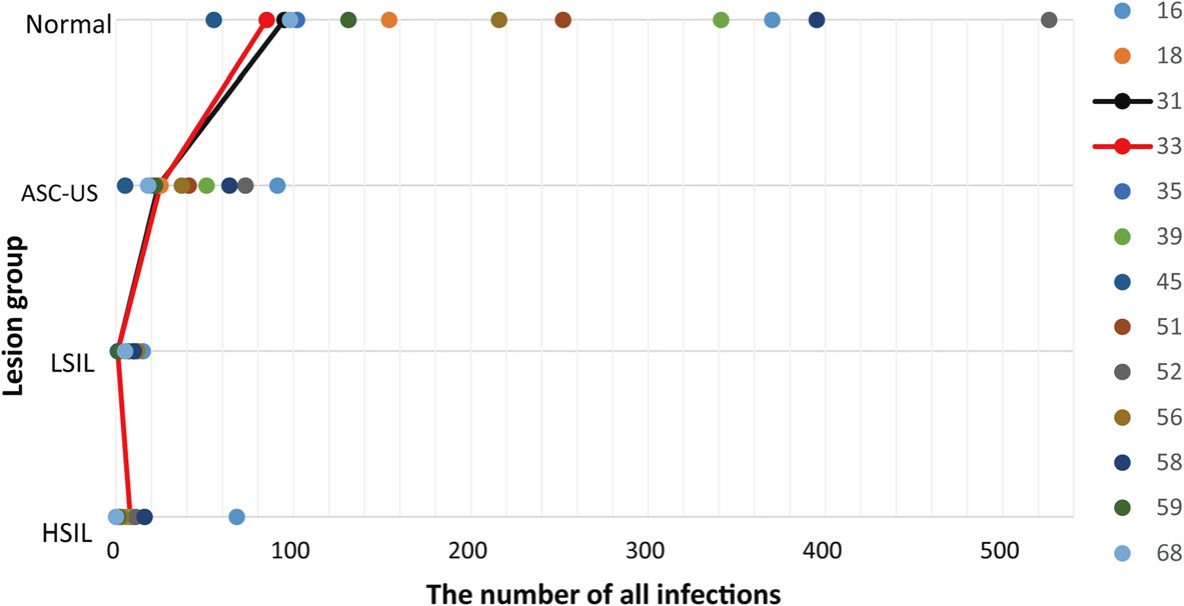
Clustering results of all infections for the 13 HR-HPV genotypes

**Fig. 5:**
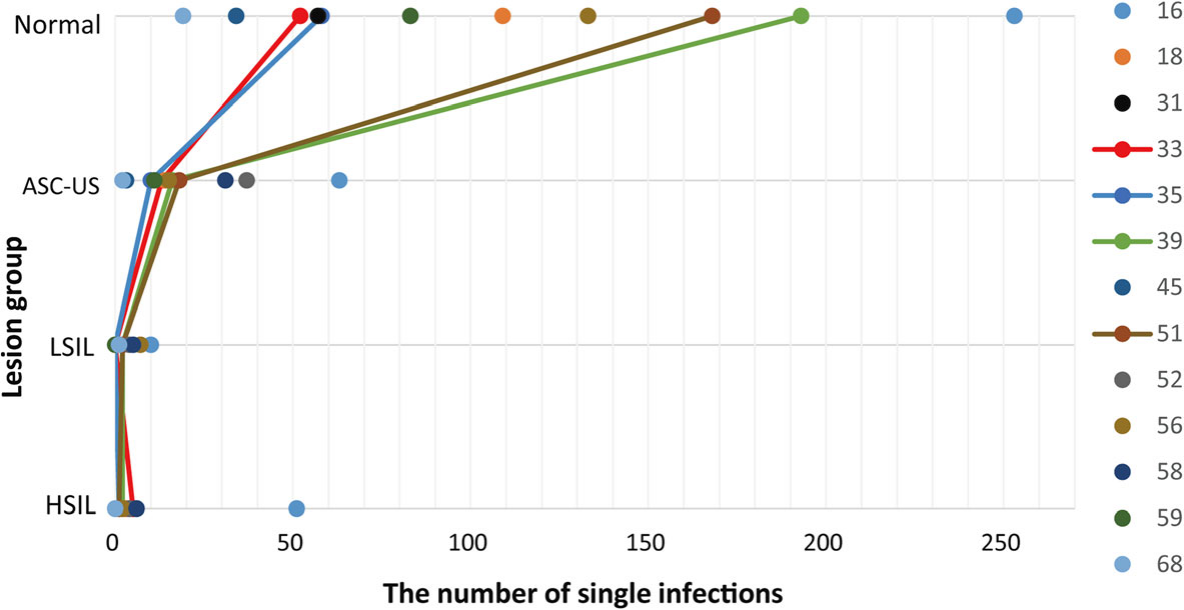
Clustering results of single infections for the 13 HR-HPV genotypes

For all infections, Fig. 4 demonstrates that the infection trends of HPV31 and HPV33 are similar in the four precancerous stages. Regarding single infections, Fig. 5 shows that not only HPV39 and HPV51 but also HPV33 and HPV35 have similar infection trends in the 4 precancerous stages.

### The impact of HR-HPV genotypes and precancerous stages on infection

**Fig. 6:**
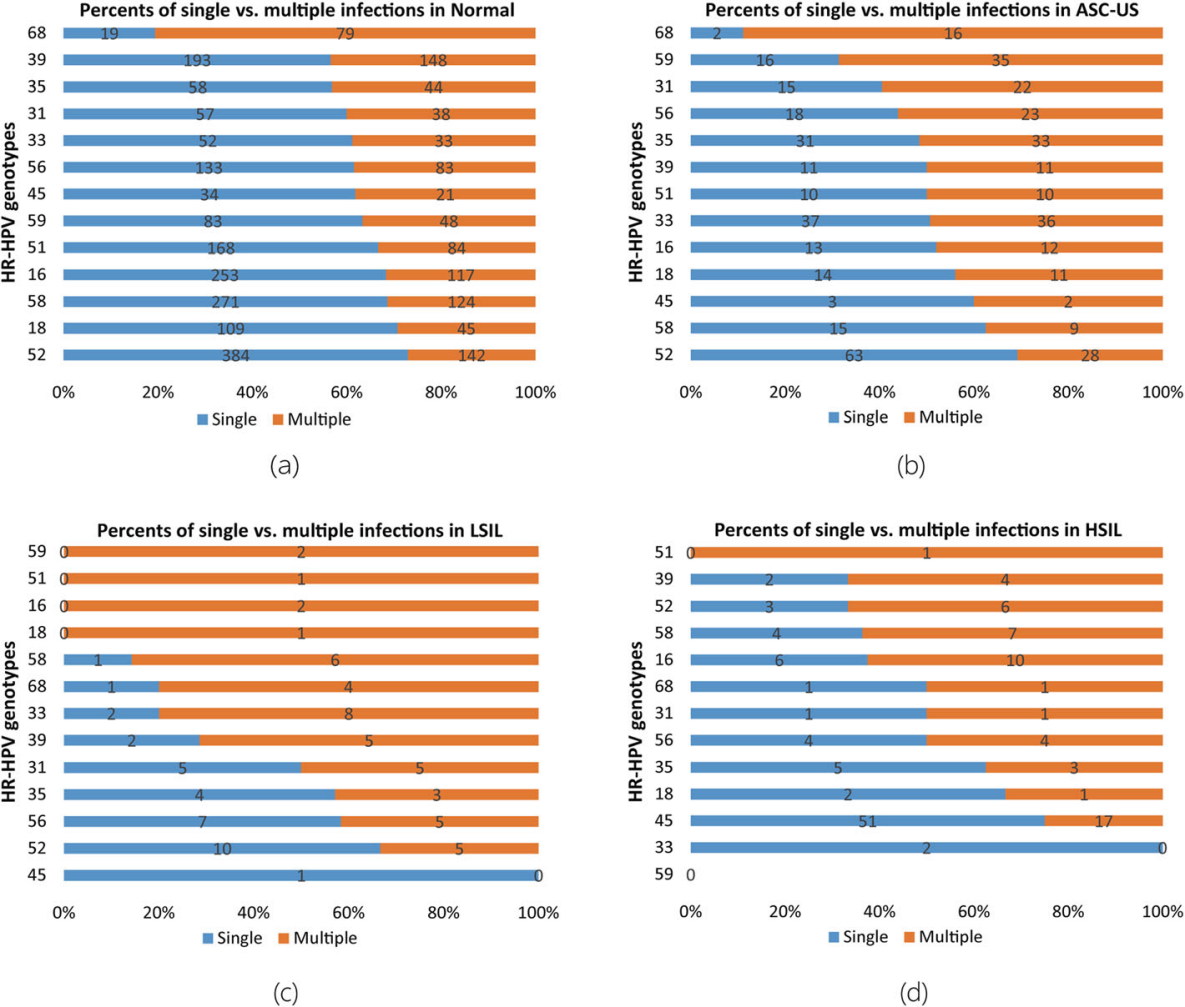
13 HR-HPV genotypes in single infections vs. multiple infections under four different precancerous stages (Normal, ASC-US, LSIL and HSIL)

Figure 6 describes the proportion of single and multiple infections for each HR-HPV genotype in different precancerous stages. Figure 6a demonstrates that the proportion of multiple infections for 12 HR-HPV genotypes is less than their single infection except for HPV68 under Normal stage. Figure 6b demonstrates that the proportion of multiple infections for 6 HR-HPV genotypes (HPV52, HPV58, HPV45, HPV18, HPV16 and HPV33) is less than their single infection under ASC-US stage. Figure 6c demonstrates that the proportion of multiple infections for 4 HR-HPV genotypes (HPV35, HPV56, HPV52 and HPV45) is less than their single infection under LSIL stage. Figure 6d demonstrates that the proportion of multiple infections for 4 genotypes (HPV35, HPV18, HPV45 and HPV33) is less than their single infection under HSIL stage. Next, the Poisson regression analysis demonstrates that only the precancerous stages are statistically significant, while the HR-HPV genotypes are not (Table 2).

**Table 2:** Parameter estimation for Poisson regression

Parameters	Df	Sum Sq	Mean Sq	F value	Pr(>F)
Genotypes	25	68,914	2757	1.58	0.0672
Precancerous	3	196,329	65,443	37.51	6.49e-15*
Residuals	75	130,868	1745		

***Note.*** ^*^Pr < 0.05

## Discussion

Since Fig. 2 shows that current total prevalence rate of the 13 HR-HPV types is less than the previous, we consider that the prevalence of both single and multiple infections was decreasing during these years in China. Additionally, since Fig. 3 shows that the prevalence rates of these HR-HPV types are inversely proportional to the severity of cervical lesions, we consider that most patients infected with HR-HPV types are in the early lesion stage (especially the squamous epithelial cells were still in the Normal stage). Thus, we have plenty of room to reduce the prevalence of HR-HPV types in China and should pay more attention to promoting cervical screening and HPV vaccine research.

Furthermore, both previous studies [3, 13] and Fig. 3 indicate that the top three greatest HR-HPV types threating to China are HPV52, HPV58 and HPV16. Figure 3 also demonstrates that neither the proportion of the 13 HR-HPV types in the same precancerous stage nor the infection rate in different stages for the same HR-HPV type is similar, which implies that the infection of different HR-HPV types in different pathological stages is not consistent. Therefore, it is better to describe the phenomenon of HR-HPV infections in different precancerous stages and HR-HPV types, but not using the overall prevalence rate of HR-HPV types for each precancerous stage [12]. Moreover, the blue part in Fig. 3 indicates that the prevalence rates of HPV16, HPV58, HPV52 and HPV18 are greater than those of other types under the HSIL stage which are easily transformed into cervical cancer.

For cluster analysis, Fig. 4 demonstrates that the infection trends of HPV31 and HPV33 in the four precancerous stages are very similar in all infections. For single infection, Fig. 5 shows that the infection trends of HPV33 and HPV35 in the four precancerous stages are very similar. Since Villiers et al. [28] previously reported that high-risk subtypes such as HPV31, HPV33, HPV35, HPV52, HPV16 and HPV58 belong to alpha-papillomavirus ninth species, our results show that such HR-HPV subtypes with common biological properties could have similar infection rate trends in precancerous stages. As we described previously, the Normal, ASC-US, LSIL and HSIL are in a disease development in order respectively. Figure 6 implies that as the disease development of precancer evolves, defense against HPV infection breaks, meanwhile, the potential of more HPV infection increases, which results in increase of multiple infection of HPV. In addition, only the precancerous stages are statistically significant when considering the impact of HR-HPV genotypes and precancerous stages on infection by regression analysis (Table 2).

## Conclusions

In conclusion, the overall prevalence rate of the 13 HR-HPV types (16.64%) is less than the previous, which results from the efforts to popularize knowledge of the high-risk HPV types and cervical cancer in recent years as well as the efforts to openly provide the HPV vaccine injection in China. However, HPV52, HPV58 and HPV16 still have the greatest impact on the health of women in China. Therefore, we should pay close attention to them through vaccine prevention and HPV genotype screening and treatment. And we consider that HPV52, HPV58 and HPV16 play a guiding role in reducing the prevalence rates of high-risk HPV types in China. In addition, we show that HR-HPV subtypes with common biological properties have similar infection rate trends in precancerous stages, and the impact of HR-HPV genotypes and precancerous stages on infection. Moreover, the single/multiple infection proportions of HPV demonstrate a trend that the multiple infections proportion of HPV increases as the disease develops.

Although we obtained several interesting new findings, this study still has many limitations. For example, because the occurrence frequency of multiple infections is significantly affected by various factors [16, 17], the findings of multiple infections can only be used as a reference. Moreover, since we lack the related molecular data, the biological mechanism of multiple infections between HR-HPV types and the related time series analysis, survival, genome and signaling pathway analysis [29–32] remain to be studied in our future research.

## Supplementary information

**Additional file 1.** Collected Datasets. This file includes three datasheets about HR-HPV data from all infections, single infections and each HR-HPV type in each lesion group.

## Data Availability

The datasets supporting the conclusions of this article are included within the article and the additional file.

## Abbreviations

HR-HPV: High-risk human papillomavirus
HPV: Human papillomavirus
LCT: Liquid-based cytology test
TCT: ThinPrep cytology test
ASC-US: Atypical squamous cells of undetermined significance
LSIL: Low-grade squamous intraepithelial lesions
HSIL: High-grade squamous intraepithelial lesions
